# Functional Roles of Metabolic Intermediates in Regulating the Human Mitochondrial NAD(P)^+^-Dependent Malic Enzyme

**DOI:** 10.1038/s41598-019-45282-0

**Published:** 2019-06-24

**Authors:** Ju-Yi Hsieh, Wan-Ting Shih, Yu-Hsuan Kuo, Guang-Yaw Liu, Hui-Chih Hung

**Affiliations:** 1 Department of Life Sciences, National Chung Hsing University, Taichung, Taiwan; 2 Institute of Biochemistry, Microbiology & Immunology, Chung Shan Medical University, Taichung, Taiwan; 3 Division of Allergy, Immunology, and Rheumatology, Chung Shan Medical University Hospital, Taichung, Taiwan; 4 Institute of Genomics and Bioinformatics, National Chung Hsing University, Taichung, Taiwan; 5 iEGG & Animal Biotechnology Center, National Chung Hsing University, Taichung, Taiwan

**Keywords:** Enzyme mechanisms, Thermodynamics

## Abstract

Human mitochondrial NAD(P)^+^-dependent malic enzyme (m-NAD(P)-ME) has a dimer of dimers quaternary structure with two independent allosteric sites in each monomer. Here, we reveal the different effects of nucleotide ligands on the quaternary structure regulation and functional role of the human m-NAD(P)-ME exosite. In this study, size distribution analysis was utilized to investigate the monomer-dimer-tetramer equilibrium of m-NAD(P)-ME in the presence of different ligands, and the monomer-dimer (*K*_d,12_) and dimer-tetramer (*K*_d,24_) dissociation constants were determined with these ligands. With NAD^+^, the enzyme formed more tetramers, and its *K*_d,24_ (0.06 µM) was 6-fold lower than the apoenzyme *K*_d,24_ (0.34 µM). When ATP was present, the enzyme displayed more dimers, and its *K*_d,24_ (2.74 µM) was 8-fold higher than the apoenzyme. Similar to the apoenzyme, the ADP-bound enzyme was present as a tetramer with a small amount of dimers and monomers. These results indicate that NAD^+^ promotes association of the dimeric enzyme into tetramers, whereas ATP stimulates dissociation of the tetrameric enzyme into dimers, and ADP has little effect on the tetrameric stability of the enzyme. A series of exosite mutants were created using site-directed mutagenesis. Size distribution analysis and kinetic studies of these mutants with NAD^+^ or ATP indicated that Arg197, Asn482 and Arg556 are essential for the ATP binding and ATP-induced dissociation of human m-NAD(P)-ME. In summary, the present results demonstrate that nucleotides perform discrete functions regulating the quaternary structure and catalysis of m-NAD(P)-ME. Such regulation by the binding of different nucleotides may be critically associated with the physiological concentrations of these ligands.

## Introduction

Malic enzyme (ME) is a homotetramer with four independent catalytic sites. This enzyme catalyzes an oxidative decarboxylation of L-malate (MAL) to pyruvate (PYR) concomitant with the reduction of NAD(P)^+^ to NAD(P)H^1,2^. The enzyme family displays conserved sequences in nature, from bacteria to mammals, indicating their importance for biological function^3^. There are three mammalian malic enzymes isoforms that are classified by their cofactor specificity and subcellular localization: cytosolic NADP^+^-dependent malic enzyme (c-NADP-ME, ME1), mitochondrial NAD(P)^+^-dependent malic enzyme (m-NAD(P)-ME, ME2), and mitochondrial NADP^+^-dependent malic enzyme (m-NADP-ME, ME3). The c-NADP-ME isoform mainly contributes to producing NADPH in the liver and adipose tissues for fatty acid biosynthesis^1,4^. Inhibition of c-NADP-ME disrupts cellular metabolism and causes cancer cells to become more vulnerable under glucose-restricted conditions^5^. The m-NADP-ME isoform distributes mainly in brain, muscle, and heart tissues. This enzyme isoform also participates in fatty acid biosynthesis and is important for insulin secretion in pancreatic *β*-cells^1,6^.

In contrast to the other two isoforms, the m-NAD(P)-ME isoform has dual cofactor specificity so that both NADH and NADPH can be generated in mitochondria by this enzyme^7^. As a result, m-NAD(P)-ME may rewire the metabolism in fast proliferating and cancer cells because it generates NADH and pyruvate as the energy source^8–10^. In addition, the enzyme generates NADPH for lipid biosynthesis and glutathione reduction that is beneficial for tumor growth^7^. Tumor cells can use glutamine and glutamate as an alternative energy source^10–12^, and m-NAD(P)-ME may play an important role in glutamine metabolism^7,10,11,13–15^. For this reason, m-NAD(P)-ME is considered a cancer drug target. Actually, m-NAD(P)-ME has been discovered to overexpress in numerous types of tumor cells, such as leukemia and melanoma, neuron, liver and breast cancer cells^9,16–18^.

The m-NAD(P)-ME and c-NADP-ME enzymes are found to associate with p53-dependent senescence, and MEs and p53 play reciprocal roles in regulating the fate of the cells^19^. In addition, both m-NAD(P)-ME knockdown and treatment with dimethyl-malate (DMM) mimicking the m-NAD(P)-ME knockdown phenotype suppress lung tumor growth *in vivo*^20^. Furthermore, m-NAD(P)-ME knockdown in human erythroleukemia cells can induce erythroid differentiation^16^. m-NAD(P)-ME also plays crucial roles in cutaneous melanoma progression^18^. The mRNA and protein expression of m-NAD(P)-ME were significantly increased, and m-NAD(P)-ME knockdown could attenuate proliferation of melanoma cells. In addition, AMPK activity, p53 levels, and the p53 downstream gene p21 are increased by depleting m-NAD(P)-ME in A375 melanoma. Embonic acid (EA) is a small-molecule inhibitor of m-NAD(P)-ME recently found from our lab. Our study demonstrated that EA suppresses the cell growth and induces the cellular senescence of the human non-small cell lung carcinoma H1299 cancer cells through inhibiting the enzyme activity of m-NAD(P)-ME^21^.

**Figure 1:**
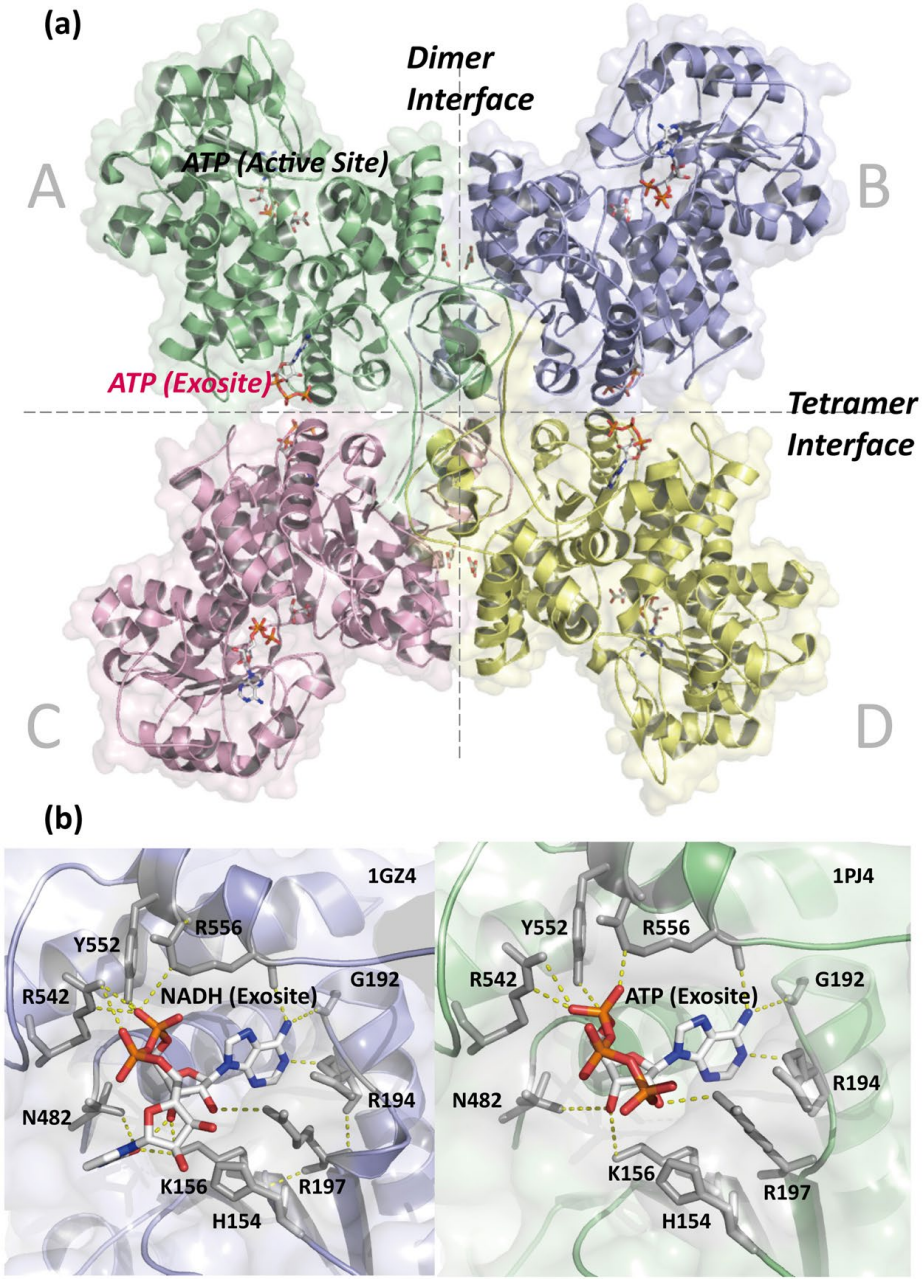
Structure and sequence alignment of human m-NADP-ME. (**a**) The crystal structure of human m-NADP-ME shows the dimer interface between the AB or CD dimers, the tetramer interface between the AD or BC dimers and the four active sites and the exosite with ATP in each monomer (PDB code: 1PJ4). (**b**) The overall binding resides of the exosite, with an NADH-binding mode (PDB code: 1PJ2) and an ATP-binding mode (PDB code: 1GZ4). These figures were generated by using PyMOL^41^.

The m-NAD(P)-ME isoform has a double-dimer structure in which the dimer interface has tighter contacts than the tetramer interface (Fig. 1a). Each subunit of m-NAD(P)-ME has two additional ligand binding sites; one is a fumarate (FUM) binding site at the dimer interface, and the other is an extra nucleotide-binding site that is bound to ATP/ADP or NAD^+^/NADH at the tetramer interface^22–24^. Human m-NAD(P)-ME has a complex kinetic regulatory mechanism and is bound to its substrate L-malate cooperatively, activated by fumarate allosterically and inhibited by ATP^23,25,26^.

To date, ten crystal structures of human m-NAD(P)-ME have been established with different kinds of ligands, including cations, substrate-analog inhibitors, the cofactor NAD^+^, the inhibitor ATP and the activator fumarate^3,22–24,27,28^. Although the overall tertiary structures of these MEs are almost identical, the conformations of the enzyme with different ligands differ considerably. Open form I, a binary complex structure with NAD^+^ alone, exposes the active site to the protein surface, whereas closed form II, a quaternary complex with NAD^+^/cation/substrate-analog or a quinary complex with ATP/cation/substrate-analog/fumarate, shields its active site from the solvent^28^.

Only the m-NAD(P)-ME isoform has two nucleotide-binding sites in each monomer; one is at the active site, the other is located at the tetramer interface, named the exosite (Fig. 1a). Sequence alignments among ME isoforms reveal that most binding residues for NAD^+^ or ATP in the exosite of m-NAD(P)-ME are not conserved in the other isoforms, suggesting that the exosite is unique to human m-NAD(P)-ME and may play a role in the enzyme regulation of m-NAD(P)-ME (Fig. 1b). The current study reveals how m-NAD(P)-ME is regulated by binding to different nucleotides in the exosite. In addition, the key residues at the exosite for ATP binding and ATP-induced dissociation of human m-NAD(P)-ME are identified. This paper also reveals that the energy metabolites (NAD^+^, ATP, ADP, malate and fumarate) regulate human m-NAD(P)-ME by altering the monomer-dimer-tetramer equilibrium, and these effects lead the enzyme to be highly controlled by the energy status of the cell.

## Materials and Methods

### Expression and purification of human m-NAD(P)-ME

The protocols for expressing and purifying m-NAD(P)-ME have been reported in our previous papers^8,22,29^. In brief, the m-NAD(P)-ME gene was subcloned into the pRH281 vector to overexpress the enzyme in *E. coli* strain BL21 with the *trp* promoter system. ATP-agarose affinity column (Sigma, St Louis, MO, USA) was used to purify the enzyme. The purified enzyme was then dialyzed by a centrifugal filter device (Amicon Ultra-15, Merck Millipore, Billerica, MA, USA) with phosphate buffer (50 mM Na_2_HPO_4_ and 2 mM *β*-mercaptoethanol, pH 7.4) or PBS (130 mM NaCl, 3 mM Na_2_HPO_4_, 7 mM NaH_2_PO_4_, and 2 mM *β*-mercaptoethanol, pH 7.4). The enzyme purity was examined by SDS-PAGE, and the protein concentration was determined by the Bradford method^30^.

### Site-directed mutagenesis

Mutagenesis of human m-NAD(P)-ME was performed using a QuikChange^TM^ kit (Agilent Technologies, Palo Alto, CA, USA) and a polymerase chain reaction (PCR) procedure with the *pfu* DNA polymerase. The primer with a specific mutation site was approximately 28–45 nucleotides in length, and the mismatched bases resided near the middle of the primer. After 18 temperature cycles, the DNA product was digested with *Dpn*I restriction enzyme (Thermo Scientific, Waltham, MA, USA) to eliminate the parental m-NAD(P)-ME plasmid. Subsequently, the digested product was transformed into the XL10-Gold^®^ *E. coli* strain (Agilent Technologies) to repair the nick. The sequence of the site-specific mutant was finally confirmed by automatic sequencing.

### Enzyme kinetic assay and analysis

The ME reaction was measured with the production rate of NADH or NADPH via the absorbance at 340 nm. The reaction mixture in a saturated condition included 1 mM NAD(P)^+^, 40 mM malate, and 10 mM MgCl_2_ in 50 mM Tris-HCl (pH 7.4). To determine the *K*_m_ and *k*_cat_ values, the concentration of one substrate was varied around its *K*_m_ values while the concentrations of the other components were fixed at saturated concentrations.

The cooperativity of malate was determined with the following equation:

$${\rm{\nu }}=\frac{{V}_{{\rm{\max }}}\times {[{\rm{Malate}}]}^{h}}{{K}_{0.5}^{h}+{[{\rm{Malate}}]}^{h}}$$

where ν is the initial velocity, V_max_ is the maximum rate of the reaction, *K*_0.5_ is the substrate concentration at half-maximal velocity, and *h* is the Hill coefficient, which represents the degree of cooperativity.

To evaluate the inhibitory effect of ATP, the initial velocity (*v*) versus [ATP] was fitted with the following equation to obtain IC_50_:

$$v=a+\frac{(b-a)}{1+{(\frac{[{\rm{ATP}}]}{{{\rm{IC}}}_{50}})}^{{\rm{C}}}}$$

where *a* and *b* represent the minimal and maximal rate of m-NAD(P)-ME, respectively. C is the slope of the curve at its midpoint. The IC_50_ value represents ATP concentration met for inhibition of enzyme activity to 50%. All of the calculations were performed using the Sigma Plot 10.0 software program (Jandel Scientific, San Rafael, CA, USA).

### Quaternary structure analysis by analytical ultracentrifugation (AUC)

Size distribution of the monomer-dimer-tetramer equilibrium of the enzyme was monitored using a Beckman Optima XL-A analytical ultracentrifuge instrument. A protein sample (380 µl) and reference buffer (400 µl) were loaded into a well-assembled cell with a double-sector centerpiece. Experiments were performed with an An-50Ti rotor at 42,000 rpm and 20 °C for 3 to 4 hours. The scans for sedimentation velocity data were monitored by the UV absorbance at 280 nm with a 480 s interval time and a 0.002 cm step size in continuous distribution mode. Using the SEDFIT 9.4c software^31–34^, the size distribution of the enzyme was analyzed with a resolution corresponding to N = 200 to cover the range of sedimentation coefficient (S) values from approximately 0.1 to 20, a confidence level of p = 0.95, and a best-fit average anhydrous frictional ratio (*f*/*f*_0_). The dissociation constants of monomer-dimer-tetramer self-association model were globally analyzed by using the SEDPHAT software.

The dissociation constants, *K*_d,12_ (between monomer and dimer) and *K*_d,24_ (between dimer and tetramer) were determined using the sedimentation data in PBS or 50 mM phosphate buffer with three protein concentrations of the enzyme complexed with different ligands (0.2 mM NAD^+^, 0.5 mM ATP, 10 mM MgCl_2_, 20 mM malate, 2 mM fumarate or 1 mM ADP). All sedimentation velocity data were globally fitted by a monomer-dimer-tetramer equilibrium model by the software program of SEDPHAT^34^. The parameters protein partial specific volume, solvent density and viscosity were calculated with the SEDNTERP software program^35^.

### Quaternary structure analysis by the native gel experiments

In the native gel electrophoresis, 8 μg of m-NAD(P)-ME proteins was first incubated with NAD^+^, ATP, and ADP, then loaded into a 3–12% Bis-Tris gradient gel (NativePAGE^TM^ Novex^®^, Life Technologies). The native gel was running under 110 Voltage with a running buffer (25 mM Tris-HCl and 192 mM glycine, pH8.3) at 4 °C. After electrophoresis, protein was detected by staining with the Coomassie brilliant blue R-250.

## Results

### Ligand-induced quaternary structure regulation of human m-NAD(P)-ME

**Figure 2:**
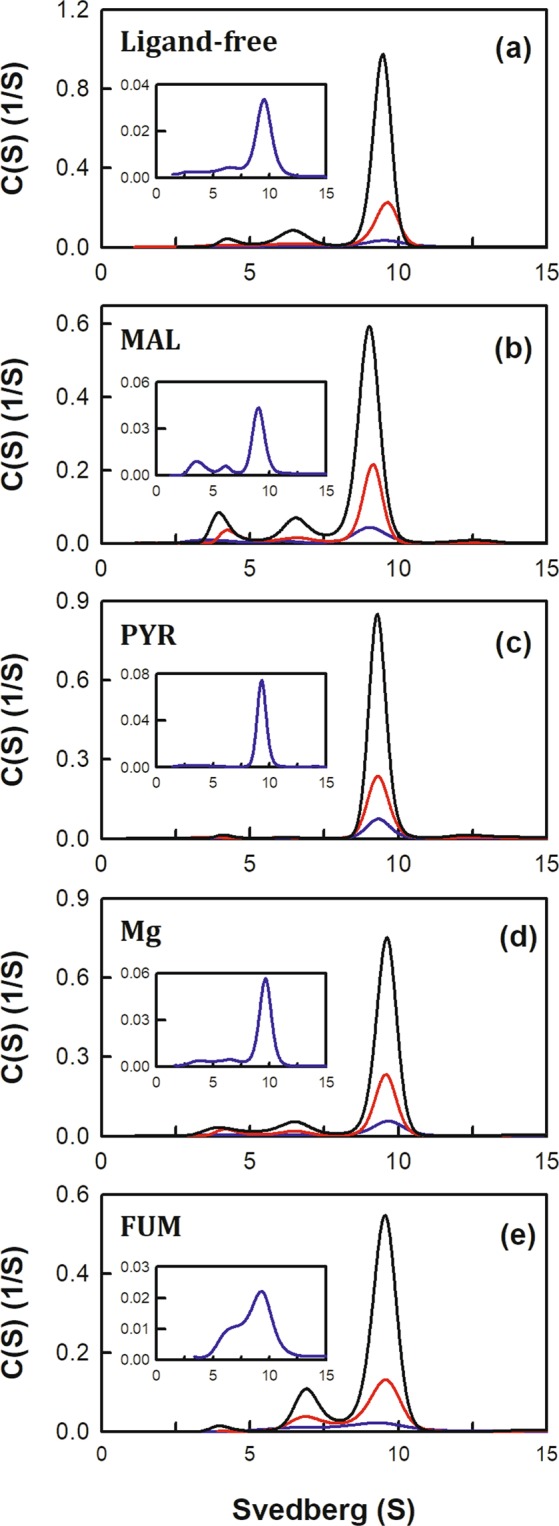
Continuous sedimentation coefficient distribution of human m-NAD(P)-ME with non-nucleotide ligands. The enzyme concentrations were 1.6, 4.7, and 14.2 μM in the phosphate buffer. (**a**) Apoenzyme; (**b**) L-malate (20 mM); (**c**) pyruvate (8 mM); (**d**) MgCl_2_ (2 mM); and (**e**) fumarate (0.2 mM).

**Figure 3:**
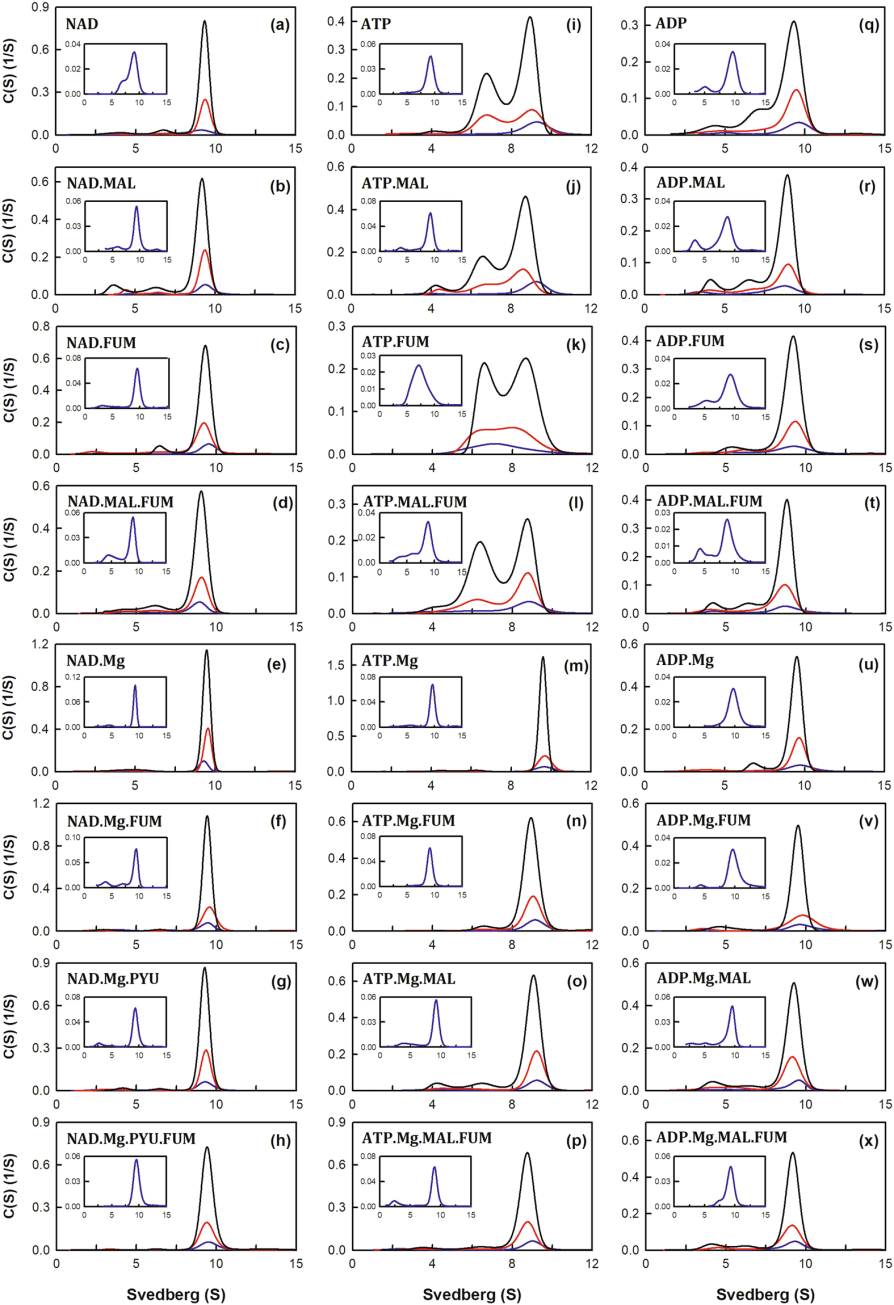
Continuous sedimentation coefficient distribution of m-NAD(P)-ME with different nucleotide ligands. The enzyme concentrations were 1.6, 4.7, and 14.2 μM in the phosphate buffer with L-malate (20 mM), pyruvate (8 mM), MgCl_2_ (2 mM) or fumarate (0.2 mM). (**a**–**h**) with NAD^+^ (0.4 mM); (**i**–**p**) with ATP (0.5 mM); and (**q**–**x**) ADP (with 1 mM).

The complex of m-NAD(P)-ME with malate (substrate), Mg^2+^ (catalytic divalent ion), NAD^+^ (nucleotide substrate), ATP or ADP (nucleotide inhibitor), fumarate (allosteric activator), and pyruvate (product) was examined for quaternary structure changes using the XL-A analytical ultracentrifuge (Figs 2 and 3). The m-NAD(P)-ME structure displayed a dimer of dimers conformation with monomer-dimer and dimer-tetramer equilibria; the sedimentation coefficients of the monomer, dimer and tetramer were approximately 4.2 S, 6.9 S and 9.3 S, respectively. Here, we demonstrated that different ligands had profound effects on the monomer-dimer and dimer-tetramer equilibria of the enzyme. The monomer-dimer (*K*_d,12_) and dimer-tetramer (*K*_d,24_) dissociation constants in the presence of different ligands are shown in Table 1.

**Table 1:** Dissociation constants for the monomer-dimer-tetramer equilibrium of human m-NAD(P)-ME in the presence of different ligands.

Without nucleotide			With NAD		
m-NAD(P)-ME	*K*_d,12_^a^ (µM)	*K*_d,24_^b^ (µM)	m-NAD(P)-ME	*K*_d,12_^a^ (µM)	*K*_d,24_^b^ (µM)
Ligand-free	0.61 ± 0.001	0.34 ± 0.001	NAD	0.34 ± 0.001	0.06 ± 1.6 × 10^−4^
MAL	2.69 ± 0.01	0.15 ± 0.001	NAD.MAL	2.92 ± 0.01	0.06 ± 3.3 × 10^−4^
PYR	0.31 ± 0.002	0.002 ± 1.4 × 10^−5^	NAD.FUM	0.01 ± 4.0 × 10^−5^	0.07 ± 1.6 × 10^−5^
Mg	0.99 ± 0.002	0.13 ± 0.001	NAD.MAL.FUM	1.47 ± 5.6 × 10^−4^	0.17 ± 2.6 × 10^−4^
FUM	0.01 ± 2.3 × 10^−5^	0.78 ± 0.002	NAD.Mg	0.04 ± 2.7 × 10^−4^	0.07 ± 4.5 × 10^−4^
			NAD.Mg.FUM	0.04 ± 9.5 × 10^−5^	0.02 ± 2.7 × 10^−4^
			NAD.Mg.PYR	0.01 ± 2.7 × 10^−5^	0.01 ± 3.5 × 10^−5^
			NAD.Mg.PYR.FUM	3 × 10^−3^ ± 1.2 × 10^−5^	0.05 ± 1.8 × 10^−4^
**With ATP**			**With ADP**		
ATP	0.06 ± 0.001	2.74 ± 0.03	ADP	0.57 ± 0.003	0.60 ± 0.003
ATP.MAL	0.51 ± 0.01	1.75 ± 0.02	ADP.MAL	1.04 ± 0.003	0.41 ± 0.001
ATP.FUM	0.02 ± 1.7 × 10^−4^	5.60 ± 0.05	ADP.FUM	0.01 ± 6.0 × 10^−5^	0.10 ± 4.8 × 10^−4^
ATP.MAL.FUM	0.10 ± 0.001	4.68 ± 0.05	ADP.MAL.FUM	0.91 ± 0.01	0.22 ± 0.002
ATP.Mg	0.03 ± 2.1 × 10^−4^	0.05 ± 3.1 × 10^−4^	ADP.Mg	0.04 ± 2.5 × 10^−4^	0.04 ± 3.3 × 10^−4^
ATP.Mg.FUM	0.02 ± 1.7 × 10^−4^	0.06 ± 0.001	ADP.Mg.FUM	0.03 ± 2.1 × 10-4	0.04 ± 0.002
ATP.Mg.MAL	0.60 ± 0.01	0.17 ± 0.002	ADP.Mg.MAL	1.88 ± 0.01	0.10 ± 5.0 × 10^−4^
ATP.Mg.MAL.FUM	0.38 ± 0.002	0.11 ± 0.001	ADP.Mg.MAL.FUM	2.79 ± 0.01	0.05 ± 2.0 × 10^−4^

^a^*K*_d,12_ indicates the monomer-dimer dissociation constant; ^b^*K*_d,24_ indicates the dimer-tetramer dissociation constant. FUM is the abbreviation of fumarate. PYR is the abbreviation of pyruvate. MAL is the abbreviation of L-malate.

### Different nucleotides have distinct effects on the tetrameric stability of the enzyme

Without any ligands, the apoenzyme was generally present as a tetramer with a few dimers and monomers; the *K*_d,12_ and *K*_d,24_ values were 0.61 and 0.34 µM, respectively (Fig. 2a). Nucleotides, including NAD^+^, ATP and ADP, had distinctive effects on the tetrameric stability of the enzyme (Fig. 3a,i,q, respectively). The NAD^+^-bound enzyme had more tetramers with a *K*_d,24_ of 0.06 µM (Fig. 3a), which was 6-fold lower than that of the apoenzyme (*K*_d,24_ of 0.34 µM, Fig. 2a; Table 1). The ATP-bound enzyme displayed more dimeric forms with a *K*_d,24_ value of 2.74 µM (Fig. 3i), which was 8-fold larger than that of apoenzyme (Table 1). The effect of ADP was less than that of ATP; the ADP-bound enzyme was present a tetramer with a small amount dimers and monomers with *K*_d,12_ and *K*_d,24_ values of 0.57 and 0.60 µM, respectively (Fig. 3q), which were similar to the apoenzyme value (Table 1). These results indicate that NAD^+^ promotes association of the dimeric enzyme into tetramers, whereas ATP stimulates the dissociation of the tetrameric enzyme into dimers, and ADP has little effect on the tetrameric stability of the enzyme. We also performed the native gel electrophoresis experiments to examine the dimer-tetramer distribution of the enzyme with NAD^+^, ATP, and ADP (Fig. S1). It was clear that the enzyme with ATP showed more dimeric forms than the tetrameric forms (lanes 3 and 7), and the ATP-bound enzyme displayed a tetrameric form significantly less than the apo-enzyme (lanes 1 and 5), the NAD^+^-bound (lanes 2 and 6), and the ADP-bound enzymes (lanes 4 and 8). In contrast to the ATP-bound enzyme, the NAD^+^-bound enzyme showed most tetrameric forms as compared to the apo-enzyme, the ATP-bound, and the ADP-bound enzymes. The native gel data coincide with the size-distribution analysis of the enzyme with ATP and NAD^+^, which states ATP and NAD^+^ induce an opposite direction in dissociation-association of the human m-NAD(P)-ME.

Notably, with binding of the substrate malate, the enzyme clearly formed monomers, but binding of pyruvate stabilized the tetrameric structure of the enzyme. The malate-bound enzyme had *K*_d,12_ and *K*_d,24_ values of 2.69 µM and 0.15 µM (Fig. 2b), while the pyruvate-bound enzyme had *K*_d,12_ and *K*_d,24_ values of 0.31 and 0.002 µM, respectively (Fig. 2c). Binding of Mg^2+^ to the enzyme seemed to favor tetramer formation by decreasing the *K*_d,24_ value from 0.34 µM to 0.13 µM (Table 1; Fig. 2d), while binding of fumarate seemed to stabilize the tetrameric structure of the enzyme by decreasing the *K*_d,12_ value from 0.61 µM to 0.01 µM (Table 1; Fig. 2e).

### Series of NAD^+^-bound enzymes

In the presence of malate, the NAD^+^-bound enzyme displayed tetramers with some monomers; the NAD^+^-malate-bound enzyme had larger *K*_d,12_ and *K*_d,24_ values (2.92 µM and 0.06 µM, respectively; Fig. 3b) than the NAD^+^-bound enzyme (0.34 µM and 0.06 µM, respectively; Fig. 3a). In contrast to the NAD^+^-malate-bound enzyme, the NAD^+^-fumarate-bound enzyme displayed more stable tetramers than the NAD^+^-bound enzyme, with reduced *K*_d,12_ and *K*_d,24_ values of 0.01 and 0.07 µM, respectively (Fig. 3c; Table 1). Interestingly, binding of malate to the NAD^+^-fumarate-bound enzyme did not further stabilize the tetrameric structure (Fig. 3d); the *K*_d,12_ and *K*_d,24_ values of the NAD^+^-malate-fumarate-bound enzyme were 1.47 and 0.17 µM, respectively (Table 1).

Binding of Mg^2+^ to the NAD^+^-bound enzyme further decreased the *K*_d,12_ values, stabilizing the tetrameric structure of the enzyme (Fig. 3e); the *K*_d,12_ value of the NAD^+^-Mg^2+^-bound enzyme was 0.04 µM, which was considerably lower than that of the NAD^+^-bound enzyme (*K*_d,12_ = 0.34 µM; Fig. 3a). Binding of fumarate to the NAD^+^-Mg^2+^-bound enzyme further stabilized the tetrameric form of the enzyme; the NAD^+^-Mg^2+^-fumarate-bound enzyme had a lower *K*_d,24_ value (0.02 µM; Fig. 3f) than the NAD^+^-Mg^2+^-bound and NAD^+^-fumarate-bound enzymes (0.07 µM for both enzyme complexes; Table 1)

Binding of pyruvate to the NAD^+^-Mg^2+^-bound enzyme further increased the dimerization and tetramerization (Fig. 3g); the *K*_d,12_ and *K*_d,24_ values of the NAD^+^-Mg^2+^-pyruvate-bound enzyme were both 0.01 µM, which were 4- and 7-fold lower than those of the NAD^+^-Mg^2+^-bound enzyme (*K*_d,12_ = 0.04 µM and *K*_d,24_ = 0.07 µM, Table 1). Binding of fumarate to the NAD^+^-Mg^2+^-pyruvate-bound enzyme further increased the quaternary structure stability of the enzyme. Of these ligand-bound enzyme complexes, the NAD^+^-Mg^2+^-pyruvate-fumarate-bound enzyme (Fig. 3h) had the lowest *K*_d,12_ value; the *K*_d,12_ value of the quinary complex was 0.003 µM, which was lower than those of the NAD^+^-Mg^2+^-pyruvate-bound and NAD^+^-Mg^2+^-fumarate-bound enzymes (0.01 µM and 0.04 µM, respectively, Table 1).

### Series of ATP-bound enzymes

The ATP-bound enzyme exhibited more dimers with *K*_d,12_ and *K*_d,24_ values of 0.06 µM and 2.74 µM, respectively (Fig. 3i); a similar result was observed for the ATP-malate-bound enzyme, which had *K*_d,12_ and *K*_d,24_ values of 0.51 µM and 1.75 µM, respectively (Fig. 3j). Both enzymes had a *K*_d,24_ value larger than that of the apoenzyme (0.34 µM; Fig. 2a). Binding of fumarate to the ATP-bound and ATP-malate-bound enzymes caused significantly more dissociation of the enzyme (Fig. 3k,l); the ATP-fumarate-bound and ATP-malate-fumarate-bound enzymes displayed equivalent amounts of dimers and tetramers with *K*_d,12_ values of 0.02 µM and 0.1 µM and *K*_d,24_ values of 5.6 µM and 4.68 µM, respectively (Table 1).

Interestingly, binding of Mg^2+^ to the ATP-bound, ATP-fumarate-bound and ATP-malate-bound enzymes largely helped to stabilize the enzyme tetramerization by decreasing the *K*_d,24_ values. Unlike the ATP-bound, ATP-fumarate-bound and ATP-malate-bound enzymes that had significant dimer levels at equilibrium (*K*_d,24_ values: 2.74 µM, 5.6 µM, 1.75 µM, respectively, Table 1), the ATP-Mg^2+^-bound, ATP-Mg^2+^-fumarate-bound and ATP-Mg^2+^-malate-bound enzymes displayed stable tetramers with low *K*_d,24_ values of 0.05 µM, 0.06 µM, 0.17 µM, respectively (Fig. 3m–o, respectively).

The effect of Mg^2+^ was also observed by comparing the ATP-malate-fumarate-bound and ATP-Mg^2+^-malate-fumarate-bound enzymes; the former (without Mg^2+^) displayed equivalent amounts of dimer and tetramers with a *K*_d,24_ value of 4.68 µM (Fig. 3l), and the latter (with Mg^2+^) demonstrated a stable tetrameric structure with a *K*_d,24_ value of 0.11 µM (Fig. 3p).

### Series of ADP-bound enzymes

Unlike binding to ATP, binding to ADP did not cause as significant dissociation of the enzyme. The ADP- and ADP-malate-bound enzymes were mainly tetramers with fewer dimers (Fig. 3q,r) than the ATP- and ATP-malate-bound enzymes, which exhibited remarkable dimer and tetramer levels (Fig. 3i,j). The ADP-bound and the ADP-malate-bound enzymes had *K*_d,24_ values of 0.6 µM and 0.41 µM, respectively, significantly lower than those of the ATP- and ATP-malate-bound enzymes (2.74 µM and 1.75 µM, respectively; Table 1).

Binding of fumarate to the ADP-bound and ADP-malate-bound enzymes caused the enzyme to reassociate rapidly (Fig. 3s,t). The ADP-fumarate-bound enzymes formed tetramers with a *K*_d,24_ value of 0.1 µM (Fig. 3s), 56-fold lower than that of the ATP-fumarate-bound enzymes (*K*_d,24_ = 5.6 µM; Fig. 3k); the ADP-malate-fumarate-bound enzyme also formed tetramers with a *K*_d,24_ value of 0.22 µM (Fig. 3t), 21-fold lower than that of the ATP-malate-fumarate-bound enzyme (*K*_d,24_ = 4.68 µM; Fig. 3l)

Binding of Mg^2+^ to the ADP-bound, ADP-fumarate-bound and ADP-malate-bound enzymes also facilitated tetramerization of the enzyme by decreasing the *K*_d,24_ values. The ADP-Mg^2+^-bound, ADP-Mg^2+^-fumarate-bound and ADP-Mg^2+^-malate-bound enzymes displayed stable tetramers with low *K*_d,24_ values of 0.04 µM, 0.04 µM, and 0.1 µM, respectively (Fig. 3u,v,w, respectively), compared with the *K*_d,24_ values of the ADP-bound, ADP-fumarate-bound and ADP-malate-bound enzymes, which were 0.6 µM, 0.1 µM, 0.41 µM, respectively (Fig. 3q,s,r, respectively). It was not surprising that the quinary complex of ADP-Mg^2+^-malate-fumarate-bound enzyme exhibited a tetrameric form with a *K*_d,24_ value of 0.05 µM (Fig. 3x), similar to that of the ADP-Mg^2+^-bound and ADP-Mg^2+^-fumarate-bound enzymes, which had a *K*_d,24_ value of 0.04 µM (Fig. 3u,v, respectively).

Given the above results, we suggested that NAD^+^ is a critical factor in the formation of a stable tetrameric structure of the enzyme. In contrast, ATP is disadvantageous for tetramerization of the enzyme, but ADP is much less harmful for the tetramer organization of the enzyme. The Mg^2+^ ion also plays an important role in stabilizing the tetrameric form of the enzyme. The tetramer of the Mg^2+^-free enzyme is less stable than that of the Mg^2+^-bound enzyme (Fig. 2), and binding of Mg^2+^ can decrease ATP-induced dissociation (Fig. 3). The crucial effect of Mg^2+^ on the structural integrity of m-NAD(P)-ME is also reported in c-NADP-ME under chemical denaturation conditions^36,37^.

### Effect of NAD^+^ and ATP on the quaternary structure of the WT and exosite mutant forms of human m-NAD(P)-ME

**Figure 4:**
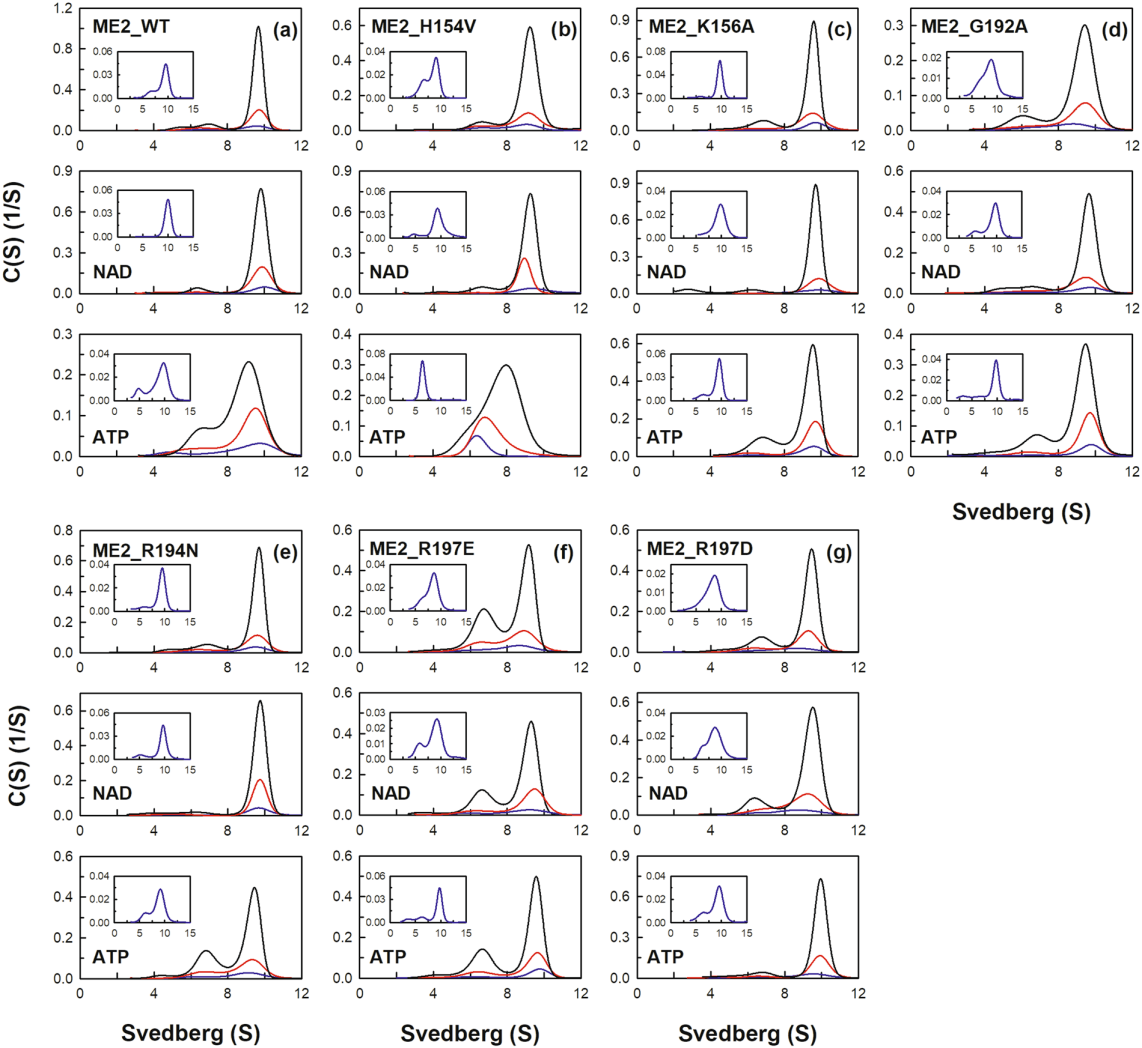
Continuous sedimentation coefficient distribution of human m-NAD(P)-ME WT and the exosite mutants. The enzyme concentrations were fixed at 1.6, 4.7, and 14.2 μM in PBS. The size distribution plots of apoenzyme (ligand-free), enzyme with NAD^+^ (0.4 mM), and enzyme with ATP (0.5 mM) were shown on the upper, middle, and lower panels, respectively. (**a**) ME2_WT; (**b**) ME2_H154V; (**c**) ME2_K156A; (**d**) ME2_G192A; (**e**) ME2_R194N; (**f**) ME2_R197E; (**g**) ME2_R197D.

**Figure 5:**
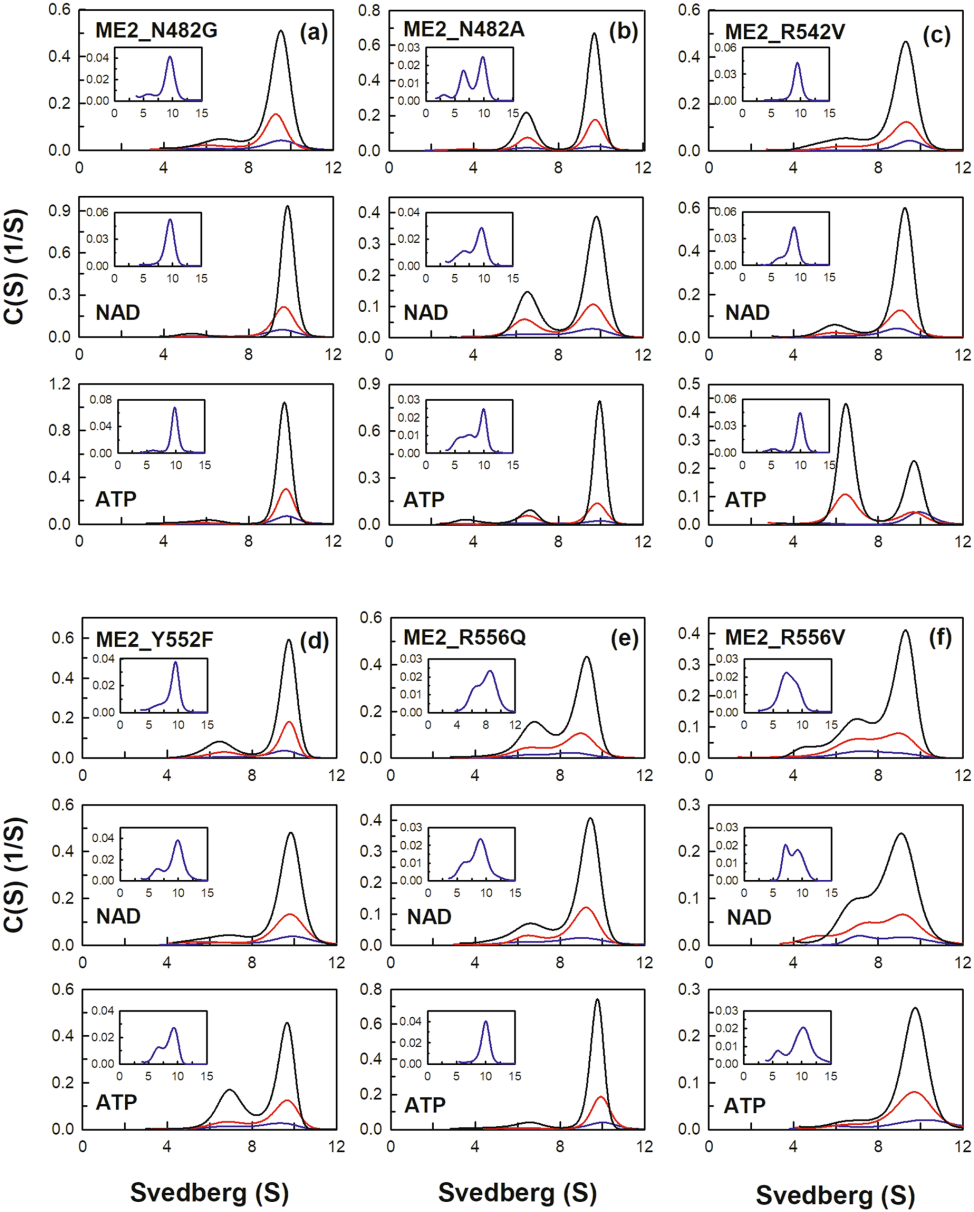
Continuous sedimentation coefficient distribution of human m-NAD(P)-ME WT and the exosite mutants. The enzyme concentrations were fixed at 1.6, 4.7, and 14.2 μM in PBS. The size distribution plots of apoenzyme (ligand-free), enzyme with NAD^+^ (0.4 mM), and enzyme with ATP (0.5 mM) were shown on the upper, middle, and lower panels, respectively. (**a**) ME2_N482G; (**b**) ME2_N482A; (**c**) ME2_R542V; (**d**) ME2_Y552F; (**e**) ME2_R556Q; (**f**) ME2_R556V.

An additional nucleotide-binding site named the exosite is located at the tetramer interface of human m-NAD(P)-ME (Fig. 1b). The exosite of the enzyme is 35 Å away from the active center, which is a binding site for ATP, ADP, and NAD^+^ ^22,23^. The crystal structures of human m-NAD(P)-ME reveal that the α-phosphate moiety of ATP interacts with residues Arg542, Tyr552, and Arg556. The ribose moiety of ATP interacts with residues Lys156, Arg197, and Asn482. The adenine moiety of ATP forms hydrogen bonds with residues Gly192, Arg194 and Arg556. (Fig. 1b). Most residues that interact with NAD^+^ are similar to those that interact with ATP. Structural data show that the exosite is uniquely found in human m-NAD(P)-ME, and the crystal structure of *Ascaris* m-NAD(P)-ME does not have this site^38^. Therefore, we further examined the function of the exosite with mutagenesis studies according to the sequence alignments among ME isoforms. These mutants complexed with NAD^+^ or ATP were analyzed with size distribution experiments (Figs 4 and 5), and the dissociation constants (*K*_d,12_ and *K*_d,24_) of the exosite mutants are shown on Table 2.

**Table 2:** Dissociation constants for the monomer-dimer-tetramer equilibrium of the exosite mutant human m-NAD(P)-ME in the presence of NAD^+^ or ATP.

m-NAD(P)-ME	Protein only		With NAD^+^		With ATP	
	*K*_d,12_^a^ (µM)	*K*_d,24_^b^ (µM)	*K*_d,12(NAD)_^c^ (µM) (*K*_d,12(NAD)_/*K*_d,12_)	*K*_d,24(NAD)_^d^ (µM) (*K*_d,24(NAD)_/*K*_d,24_)	*K*_d,12(ATP)_^e^ (µM) (*K*_d,12(ATP)_/*K*_d,12_)	*K*_d,24(ATP)_^f^ (µM) (*K*_d,24(ATP)_/*K*_d,24_)
WT	0.24 ± 0.002	0.35 ± 0.003	0.66 ± 0.01 (2.8)	0.05 ± 0.001 (0.14)	0.64 ± 0.01 (2.7)	2.72 ± 0.06 (7.8)
H154V	0.03 ± 0.002	0.46 ± 0.003	0.07 ± 0.001 (2.3)	0.16 ± 0.001 (0.3)	0.46 ± 0.01 (15.3)	4.03 ± 0.04 (8.7)
K156A	0.12 ± 0.004	0.32 ± 0.01	1.36 ± 0.05 (11.3)	0.08 ± 0.003 (0.3)	0.32 ± 0.01 (2.7)	0.80 ± 0.02 (2.5)
G192A	0.09 ± 0.002	0.30 ± 0.001	0.06 ± 0.002 (0.7)	0.18 ± 0.01 (0.6)	0.21 ± 0.01 (2.3)	0.40 ± 0.01 (1.3)
R194N	0.31 ± 0.01	0.23 ± 0.01	0.17 ± 0.01 (0.5)	0.04 ± 0.001 (0.2)	0.08 ± 0.001 (0.3)	1.44 ± 0.03 (6.3)
R197E	0.03 ± 0.001	2.33 ± 0.08	0.10 ± 0.003 (3.3)	1.11 ± 0.04 (0.5)	0.10 ± 0.003 (3.3)	0.59 ± 0.02 (0.3)
R197D	0.80 ± 0.02	0.26 ± 0.01	0.06 ± 0.003 (0.1)	0.81 ± 0.04 (3.1)	0.69 ± 0.02 (0.9)	0.11 ± 0.003 (0.4)
N482G	0.05 ± 0.01	0.18 ± 0.002	0.38 ± 0.002 (7.6)	0.04 ± 0.01 (0.2)	0.10 ± 0.003 (2.0)	0.06 ± 0.002 (0.3)
N482A	0.29 ± 0.01	0.95 ± 0.03	0.58 ± 0.03 (2.0)	0.97 ± 0.05 (1.0)	1.44 ± 0.04 (49.7)	0.35 ± 0.01 (0.4)
R542V	0.01 ± 0.001	0.44 ± 0.01	1.87 ± 0.08 (187.0)	0.21 ± 0.01 (0.5)	0.02 ± 0.001 (2.0)	9.87 ± 0.38 (22.4)
Y552F	0.05 ± 0.001	0.51 ± 0.01	1.57 ± 0.11 (31.4)	0.09 ± 0.01 (0.2)	0.01 ± 0.001 (0.2)	2.58 ± 0.07 (5.1)
R556Q	0.01 ± 0.003	1.75 ± 0.06	0.18 ± 0.01 (18.0)	0.91 ± 0.03 (0.5)	0.40 ± 0.01 (40.0)	0.18 ± 0.01 (0.1)
R556V	0.13 ± 0.001	2.30 ± 0.02	0.60 ± 0.01 (4.6)	4.07 ± 0.08 (1.8)	2.03 ± 0.06 (15.6)	0.18 ± 0.01 (0.08)

^a^*K*_d,12_ and ^b^*K*_d,24_ indicate the monomer-dimer and dimer-tetramer dissociation constants, respectively; ^c^*K*_d,12(NAD)_ and ^d^*K*_d,24(NAD)_ indicate the monomer-dimer and dimer-tetramer dissociation constants, respectively, with 0.4 mM NAD^+^ treatment; ^e^*K*_d,12(ATP)_ and ^f^*K*_d,24(ATP)_ indicate the monomer-dimer and dimer-tetramer dissociation constants, respectively, with 0.5 mM ATP treatment.

As mentioned above, m-NAD(P)-ME WT without NAD^+^ or ATP exhibited a monomer-dimer-tetramer equilibrium with *K*_d,12_ and *K*_d,24_ values of 0.24 and 0.35 µM, respectively (Fig. 4a). Among these exosite mutants, R197E, N482A, R556Q and R556V displayed more dimers at equilibrium, with *K*_d,24_ values of 2.33 µM, 0.95 µM, 1.75 µM and 2.3 µM, respectively, which were several-fold higher than the WT value (Figs 4f and 5b,e,f, respectively; Table 2), indicating that Arg197, Asn482 and Arg556 are the crucial residues to maintain the quaternary structure integrity of m-NAD(P)-ME.

Additionally, as aforementioned, m-NAD(P)-ME WT complexed with NAD^+^ or ATP exhibited a monomer-dimer-tetramer equilibrium with *K*_d,12(NAD)_ and *K*_d,24(NAD)_ values of 0.66 µM and 0.05 µM and *K*_d,12(ATP)_ and *K*_d,24(ATP)_ values of 0.64 µM and 2.72 µM, respectively (Fig. 4a). For m-NAD(P)-ME WT, NAD^+^ binding increased the tetramer formation with a decreased *K*_d,24(NAD)_ value; the ratio of *K*_d,24(NAD)_/*K*_d,24_ was 0.14. Additionally, ATP binding increased the dimer formation, with an increased *K*_d,24(ATP)_ value; the *K*_d,24(ATP)_/*K*_d,24_ was 7.8. However, most exosite mutants did not follow this pattern as obviously as WT.

For G192A, R197E, R197D, N482A, R542V, R556Q and R556V, the tetramerization increased less than for WT after NAD^+^ binding (Figs 4d,f,g and 5b,c,e,f); the *K*_d,24(NAD)_/*K*_d,24_ ratio was 0.14 for WT but more than 0.5 for these exosite mutants (Table 2). Among these mutants, R197D, N482A and R556V were more notable since the *K*_d,24(NAD)_/*K*_d,24_ ratio of these mutant enzymes was above 1, which was 10-fold larger than that of WT (0.14). These data indicated that Gly192, Arg197, Asn482, Arg542 and Arg556 are important for binding to NAD^+^ and that Arg197, Asn482, and Arg556 are responsible for NAD^+^-triggered tetramerization.

Moreover, for R197E, R197D, N482G, N482A, R556Q and R556V, the ATP-induced dissociation from tetramers to dimers was not significant (Figs 4f,g and 5a,b,e,f); the *K*_d,24(ATP)_/*K*_d,24_ ratio was 7.8 for WT but was less than 0.5 for these exosite mutants (Table 2). The ratio of *K*_d,24(ATP)_/*K*_d,24_ of R197E, R197D, N482G, N482A, R556Q and R556V was 0.3, 0.4, 0.3, 0.4, 0.1 and 0.08, respectively, which was significantly less than that of WT (7.8), indicating that the ATP-induced dissociation from tetramer to dimer did not occur in these exosite mutants. Actually, for the R197D, N482G, R556Q and R556V mutants, ATP did not cause further dissociation of the tetramer but instead tended to promote reassociation of the dimeric forms of these mutants (Figs 4g and 5a,e,f).

In summary, these data suggest that Arg197, Asn482 and Arg556 are important for the ATP binding and ATP-induced dissociation of human m-NAD(P)-ME.

### Kinetic properties of exosite mutants of human m-NAD(P)-ME

Human m-NAD(P)-ME exhibited sigmoidal kinetics with respect to malate, and the degree of cooperativity of malate evaluated by the Hill coefficient (*h*) was approximately 2 (Table 3). The enzyme was allosterically activated by fumarate, leading to a decrease in the values of *K*_0.5,malate_ and *K*_m,NAD_.

**Table 3:** Kinetic parameters of m-NAD(P)-ME WT and exosite mutants.

m-NAD(P)-ME		*K*_m,NAD_ (mM)	*K*_0.5,malate_ (mM)	*k*_cat_ (s^−1^)	*h*	IC_50,ATP_ (mM)
WT	(−)	0.54 ± 0.06	10.67 ± 0.64	252.0 ± 8.2	2.41 ± 0.32	0.20 ± 0.04
	(+)	0.39 ± 0.05	2.98 ± 0.21	261.7 ± 6.0	1.10 ± 0.09	
H154V	(−)	0.97 ± 0.06	15.31 ± 0.51	134.6 ± 2.9	1.99 ± 0.10	0.65 ± 0.03
	(+)	0.53 ± 0.04	4.94 ± 0.26	140.5 ± 2.5	1.08 ± 0.05	
K156A	(−)	0.65 ± 0.07	10.12 ± 0.21	209.9 ± 6.2	1.65 ± 0.04	0.22 ± 0.10
	(+)	0.32 ± 0.03	3.56 ± 0.40	223.6 ± 5.1	1.01 ± 0.10	
G192A	(−)	0.57 ± 0.07	11.18 ± 2.08	210.6 ± 7.7	1.30 ± 0.19	0.73 ± 0.07
	(+)	0.32 ± 0.03	2.65 ± 0.18	230.0 ± 4.4	1.19 ± 0.10	
R194N	(−)	1.60 ± 0.16	19.53 ± 0.91	204.4 ± 8.3	2.18 ± 0.15	0.50 ± 0.28
	(+)	0.76 ± 0.07	5.11 ± 0.50	227.4 ± 6.1	1.08 ± 0.08	
R197E	(−)	2.37 ± 0.28	23.37 ± 4.48	165.2 ± 20.4	1.65 ± 0.30	0.77 ± 0.28
	(+)	1.11 ± 0.09	4.55 ± 0.37	187.6 ± 5.2	1.24 ± 0.10	
R197D	(−)	0.46 ± 0.05	17.82 ± 2.01	206.8 ± 0.1	1.76 ± 0.23	0.24 ± 0.03
	(+)	0.13 ± 0.02	4.98 ± 0.28	216.0 ± 0.1	1.06 ± 0.05	
N482G	(−)	1.50 ± 0.14	23.81 ± 2.70	263.2 ± 19.7	1.59 ± 0.14	0.40 ± 0.03
	(+)	0.61 ± 0.04	4.23 ± 0.47	309.7 ± 11.5	1.00 ± 0.09	
N482A	(−)	0.70 ± 0.17	17.43 ± 5.10	169.1 ± 0.6	1.87 ± 0.70	0.14 ± 0.05
	(+)	0.02 ± 0.01	1.02 ± 0.08	215.6 ± 0.1	1.04 ± 0.14	
R542V	(−)	2.18 ± 0.27	27.00 ± 3.44	175.6 ± 14.0	1.43 ± 0.09	0.83 ± 0.21
	(+)	0.91 ± 0.18	5.89 ± 0.78	239.3 ± 12.6	1.06 ± 0.11	
Y552F	(−)	0.97 ± 0.25	17.09 ± 0.88	145.8 ± 4.5	1.61 ± 0.08	0.59 ± 0.08
	(+)	0.37 ± 0.05	4.11 ± 0.28	171.5 ± 3.4	1.05 ± 0.06	
R556Q	(−)	1.16 ± 0.22	19.01 ± 2.40	220.5 ± 16.2	1.71 ± 0.27	0.47 ± 0.04
	(+)	0.69 ± 0.06	4.75 ± 0.31	251.8 ± 5.6	1.20 ± 0.07	
R556V	(−)	1.08 ± 0.06	15.90 ± 1.51	195.2 ± 12.8	2.35 ± 0.47	0.57 ± 0.10
	(+)	0.36 ± 0.02	1.87 ± 0.26	204.9 ± 9.5	1.24 ± 0.30	
m-NAD(P)-ME						IC_50,ADP_ (mM)
WT						3.74 ± 0.16

(−) without fumarate and (+) with 5 mM fumarate.

For most exosite mutants, the malate cooperativity that was represented by the *h* value was not notably changed, and the *K*_0.5,malate_ or *K*_m,NAD_ values were still largely reduced by fumarate, indicating that these exosite mutants still exhibited cooperative and allosteric properties similar to those of the WT enzyme. In addition, even though the *k*_cat_ value of H154V and Y552F was reduced to half of the WT value, most exosite mutants retained its (or their) catalytic activity (or activities), as indicated by the *k*_cat_ values (Table 3).

A hyperbolic activation curve of human m-NAD(P)-ME was obtained from a fumarate titration series, and the maximal activation varied approximately 2-fold (Fig. S2A). Interestingly, the activating effect of fumarate on most exosite mutants was more significant than that on the WT enzyme, implying that the conformational change in the exosite was associated with the binding of fumarate at the dimer interface (Fig. 1A) and the subsequent activation of the enzyme (Fig. S2).

A previous study reported that the inhibiting effect of ATP on m-NAD(P)-ME activity occurred through competition with NAD^+^ at the catalytic site. The results of the current study demonstrated that most exosite mutants were less sensitive to ATP inhibition, with elevated IC_50,ATP_ values (Fig. S3 and Table 3), indicating that ATP binding to the exosite has some effects on enzyme inhibition. Among these exosite mutants, R542V is a special case because this mutant exhibited a predominance of the dimeric form in the presence of ATP (Fig. 5c). The dimeric R542V was less sensitive to ATP inhibition, showing the highest IC_50,ATP_ value among these mutants.

## Discussion

### Nucleotides perform discrete functions in regulating the quaternary structure and catalysis of m-NAD(P)-ME

The enzyme m-NAD(P)-ME has a dimer of dimers quaternary structure with four identical subunits, and catalysis of the enzyme is regulated by the substrate L-malate, cofactor NAD(P)^+^, divalent cation Mg^2+^, activator fumarate, and inhibitor ATP when these ligands are bound to their respective sites at the dimer or tetramer interfaces (Fig. 1a). The results of the present study demonstrate that different nucleotides have disparate effects on regulating the dynamics of the quaternary structure of m-NAD(P)-ME (Figs 2 and 3).

NAD^+^ and ATP, the nucleotide ligands of the enzyme, can bind to either the active center or the exosite, which have been demonstrated in various structures of the enzyme complex^22–24,27,28^. We have previously reported that dissociation of m-NAD(P)-ME tetramers into dimers causes the enzyme to become less active and that the tetrameric organization is essential for full catalytic efficiency of the enzyme^39^. Combining this information with the results of the present study, we suggest that NAD^+^ acts as a substrate at the active site and as an activator at the exosite, which promotes tetramer formation of the enzyme and consequently stimulates the enzyme activities. By contrast, ATP acts as an inhibitor not only by binding to the active site but also by binding to the exosite; the latter causes the enzyme to dissociate from tetramers to dimers and subsequently suppresses the enzyme activity. Intriguingly, compared with ATP, ADP has a less prominent effect of disrupting the quaternary structure of the enzyme and inhibiting the enzyme activity (Figs 2, 3 and S3). Therefore, m-NAD(P)-ME regulation occurs through the binding of different nucleotide ligands, and such regulation may be critically associated with the physiological concentrations of these ligands.

In mitochondria, m-NAD(P)-ME can be largely activated at millimolar concentrations of NAD^+^ and fumarate to trigger the conversion of malate to pyruvate, followed by the production of acetyl-CoA and finally ATP. When cells have sufficient ATP, ATP inactivates the enzyme by binding to the active site to suppress the enzyme activity and by binding to the exosite to disrupt the tetramer formation of the enzyme. Therefore, the enzyme activity, which is highly controlled by the energy status of the cell, is attributable to its high sensitivity to the ratio of [ATP]/[ADP], as well as to TCA cycle intermediates, such as fumarate and succinate^40^.

### Functional role of the exosite

Considering the size distribution analysis and mutagenesis studies of the exosite, we suggest that the exosite plays a crucial role in enzyme regulation through the binding of different nucleotides; the binding of NAD^+^ to this site promotes the formation of a stable tetramer that can perform maximal enzyme activity; by contrast, once ATP is bound to this site, the enzyme dissociates from a tetramer to dimers, leading to lesser enzyme activity.

The complicated enzyme regulation of m-NAD(P)-ME involves the quaternary structure interconversion, which occurs through the binding of different nucleotide ligands in the exosite. This complex regulation of the enzyme is quite similar to that of phosphofructokinase-1 (PFK-1). Indeed, several common features exist in m-NAD(P)-ME and PFK-1 (Table 4). Most importantly, the fully functional form for catalysis of both enzymes is the tetramer; binding of ATP to the additional nucleotide-binding site (exosite for m-NAD(P)-ME and inhibitory nucleotide-binding site for PFK-1) causes the enzyme to dissociate from a tetramer to dimers, with a subsequent loss of the enzymatic activity. Therefore, as a result of regulating the quaternary structure changes, ATP is an allosteric inhibitor of both enzymes. In addition, both enzymes are highly sensitive to energy metabolism in living cells. In the cytosol, PFK-1 activities determine the rate of glycolysis, which is the first pathway of glucose catabolism; in mitochondria, m-NAD(P)-ME catalyzes the oxidative decarboxylation of malate to pyruvate while concomitantly producing NADH. Thus, when ATP levels are sufficient or the ratio of [ATP]/[ADP] is elevated under physiological conditions, the activities of both enzymes will be suppressed until ATP is depleted.

**Table 4:** Common features of m-NAD(P)-ME and PFK-1.

Feature	m-NAD(P)-ME	PFK-1
Subcellular Location	Mitochondria	Cytosol
Quaternary Structure	Tetramer (most active) Dimer (less active)	Tetramer (most active) Dimer (less active)
Allosteric Nucleotide-binding Site	**One site** **Exosite** at the tetramer interface; can bind to NAD^+^, ATP or ADP^22–24,27,28^ **NAD**^**+**^ **binding:** Enhances the formation of a tetrameric structure **ATP binding:** Promotes the dissociation of tetramers into dimers	**Two sites** **Activating** nucleotide-binding site can bind to ADP or AMP. **Inhibitory** nucleotide-binding site can bind to ATP or ADP. **ATP binding:** Promotes the dissociation of tetramers into dimers^42,43^
Nucleotide Substrate	NAD^+^	ATP (<1 mM)
Nucleotide Inhibitor	ATP (IC_50_ = 0.2 mM) ADP (IC_50_ = 3.7 mM) tetramers disassociate into dimers	ATP (>1 mM) ADP (at mM) tetramers disassociate into dimers
Nucleotide Activator	NAD^+^	ADP (at μM)

Kinetically, ATP can inhibit the enzyme efficiently by binding to either the active site or the exosite of m-NAD(P)-ME, but ADP is not as effective as ATP. Since the enzyme is regulated directly by ATP concentrations, we suggest that m-NAD(P)-ME may play a crucial role in cellular energy metabolism.

## Supplementary information

Supporting information
