# Supplementary material for: Functional Roles of Metabolic Intermediates in Regulating the Human Mitochondrial NAD(P)+-Dependent Malic Enzyme
Source: Sci Rep. 2019 Jun 24;9:9081. doi: 10.1038/s41598-019-45282-0 (PMC6591397; doi:10.1038/s41598-019-45282-0)
Supplement: Supplementary file 1 — Supporting information [file 41598_2019_45282_MOESM1_ESM.docx]

**Supplementary Figures**

**Functional Roles of Metabolic Intermediates in Regulating the Human Mitochondrial NAD(P)^+^-Dependent Malic Enzyme**

**Ju-Yi Hsieh, Wan-Ting Shih, Yu-Hsuan Kuo, Guang-Yaw Liu and Hui-Chih Hung**

**
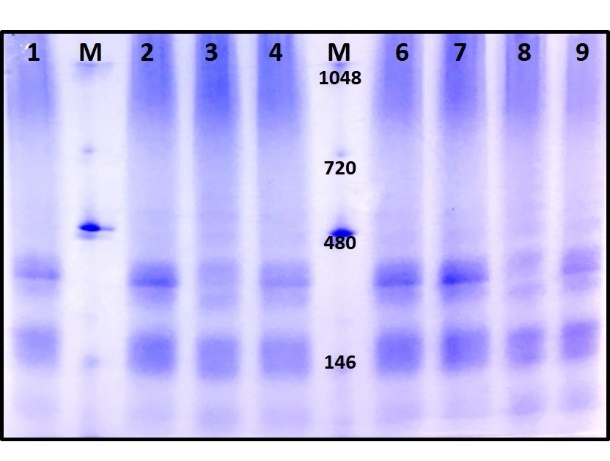
**

**Figure S1. Quaternary structure of m-NAD(P)-ME determined by the native gel electrophoresis.** The dimer-tetramer distribution of m-NAD(P)-ME was presented on the 3-12% Bis-Tris Native PAGE (NativePAGE^TM^ Novex^®^, Life Technologies). 8 µg of m-NAD(P)-ME proteins were used in the experiments. lane 5: Molecular marker (M); lane 1: Ligand-free; lane 2: with 0.4 mM NAD^+^; lane 3: with 0.5 mM ATP; lane 4: with 1 mM ADP; Lane 6: with Ligand-free; lane 7: with 2 mM NAD^+^; lane 8: with 2 mM ATP; lane 9: with 2 mM ADP. Ligand concentrations used in lanes 2-4 were similar to those in the AUC analysis, which was shown on Figure 3.

**
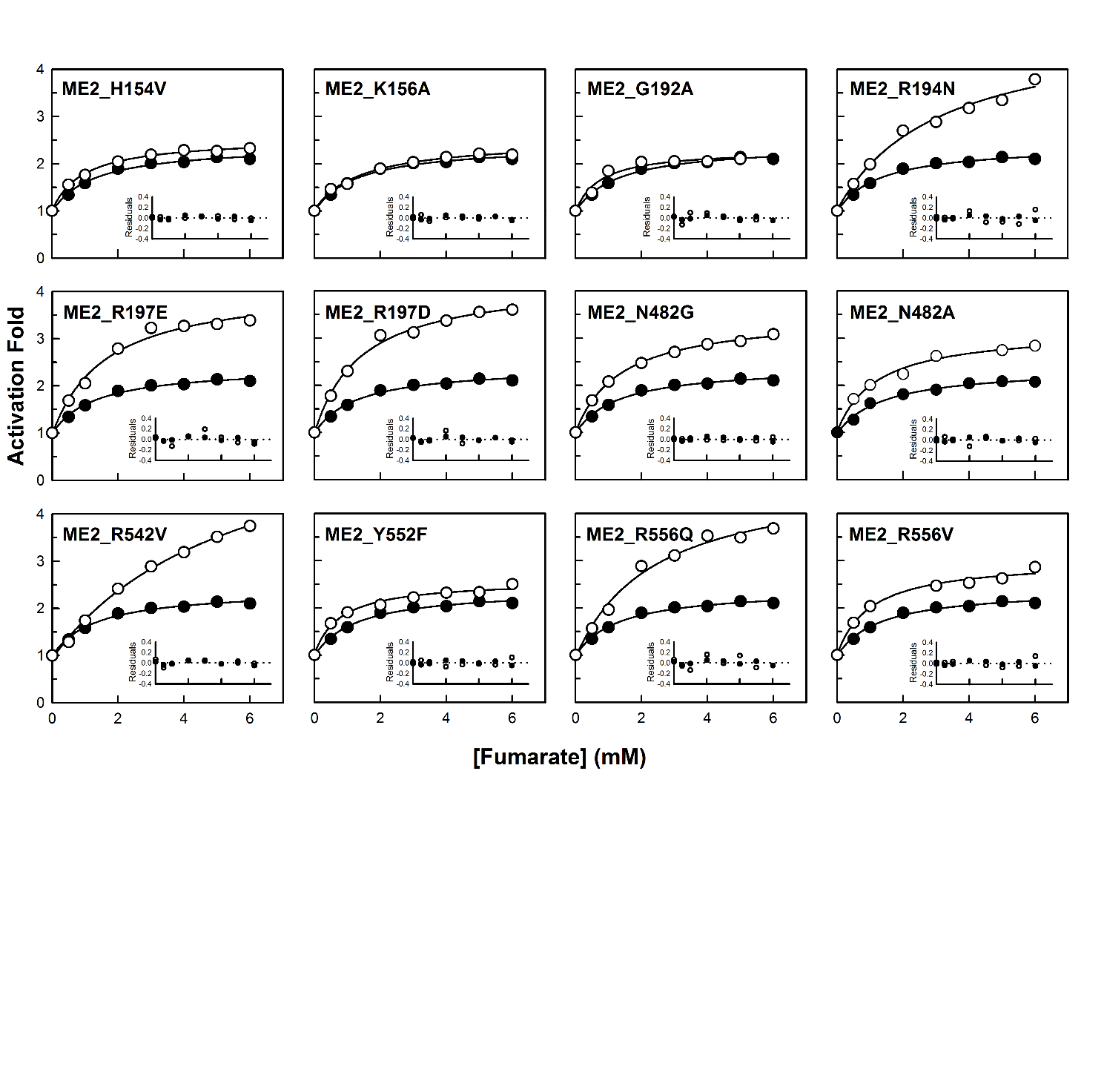
**

**Figure S2. Fumarate activating effect on human m-NAD(P)-ME WT and the exosite mutants.** The assay mixture contained 15 mM L-malate, 10 mM MgCl_2_, 1 mM NAD^+^ and 50 mM Tris-HCl (pH 7.4) with various concentrations of fumarate in the range of 0 to 6 mM.

**
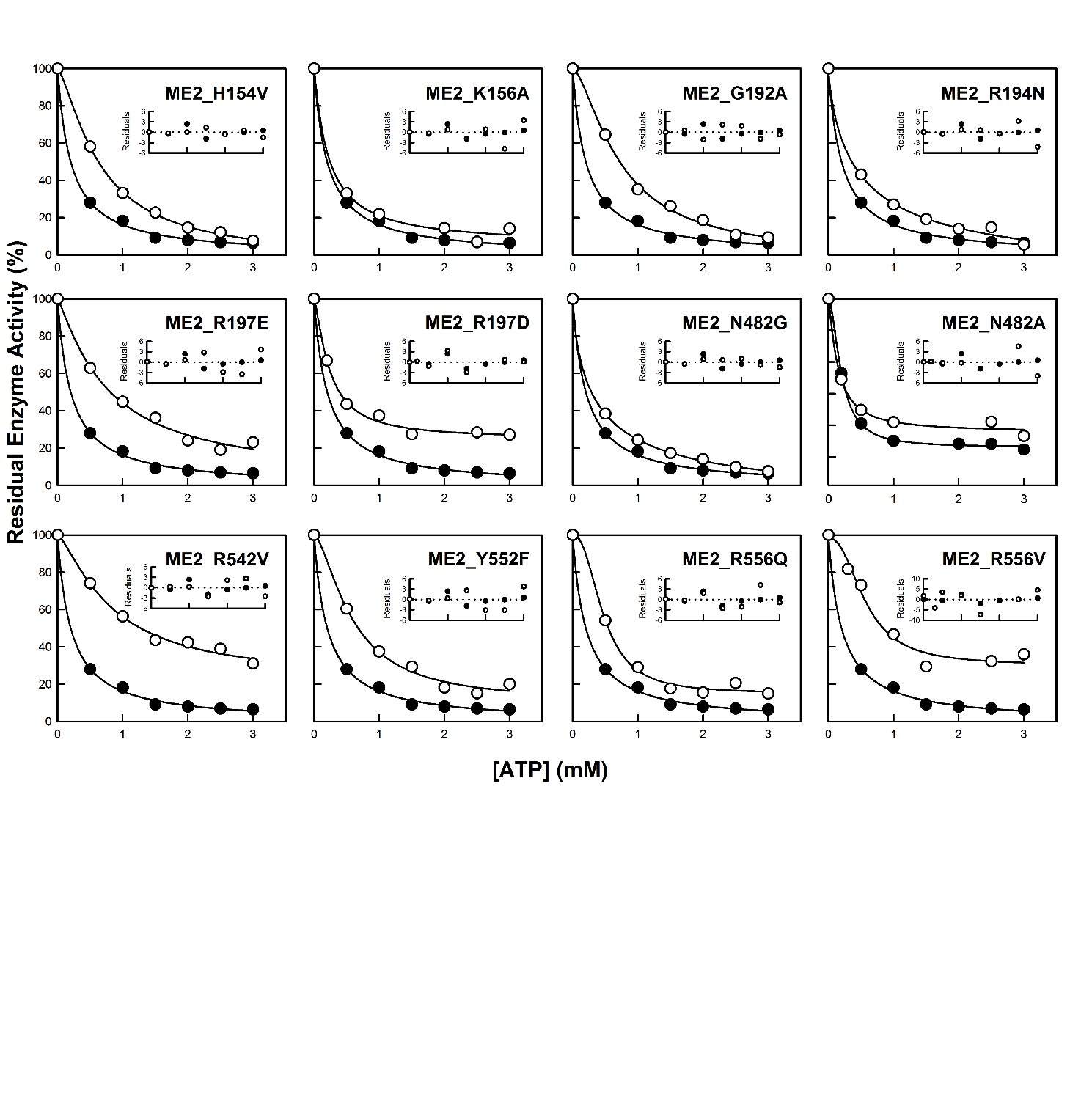
**

**Figure S3. ATP inhibition of human m-NAD(P)-ME WT and the exosite mutants.** The assay mixture contained 15 mM L-malate, 10 mM MgCl_2_, 1 mM NAD^+^ and 50 mM Tris-HCl (pH 7.4) with various concentrations of ATP in the range of 0 to 3 mM.
